# Dietary choline supplementation attenuated high-fat diet-induced inflammation through regulation of lipid metabolism and suppression of NFκB activation in juvenile black seabream (*Acanthopagrus schlegelii*)

**DOI:** 10.1017/jns.2019.34

**Published:** 2019-11-22

**Authors:** Min Jin, Tingting Pan, Douglas R. Tocher, Mónica B. Betancor, Óscar Monroig, Yuedong Shen, Tingting Zhu, Peng Sun, Lefei Jiao, Qicun Zhou

**Affiliations:** 1 Laboratory of Fish and Shellfish Nutrition, School of Marine Sciences, Ningbo University, Ningbo 315211, People's Republic of China; 2 Faculty of Natural Sciences, Institute of Aquaculture, University of Stirling, Stirling FK9 4LA, UK; 3 Instituto de Acuicultura Torre de la Sal, Consejo Superior de Investigaciones Científicas (IATS-CSIC), 12595 Ribera de Cabanes, Castellón, Spain

**Keywords:** Choline, High-fat diets, Lipid metabolism, Inflammation, NFκB, *accα*, acetyl-CoA carboxylase α, ALT, alanine aminotransferase, AST, aspartate aminotransferase, *cpt1a*, carnitine palmitoyltransferase 1a, *fas*, fatty acid synthase, HFD, high-fat diet, HFD + C1, HFD + choline (3 g/kg), HFD + C2, HFD + choline (6 g/kg), HFD + C3, HFD + choline (12 g/kg), *hsl*, hormone-sensitive lipase, LPS, lipopolysaccharide, qPCR, quantitative PCR, *srebp-1*, sterol regulatory element-binding protein-1, *tgfβ-1*, transforming growth factor β-1

## Abstract

The present study aimed to investigate whether dietary choline can regulate lipid metabolism and suppress NFκB activation and, consequently, attenuate inflammation induced by a high-fat diet in black sea bream (*Acanthopagrus schlegelii*). An 8-week feeding trial was conducted on fish with an initial weight of 8·16 ± 0·01 g. Five diets were formulated: control, low-fat diet (11 %); HFD, high-fat diet (17 %); and HFD supplemented with graded levels of choline (3, 6 or 12 g/kg) termed HFD + C1, HFD + C2 and HFD + C3, respectively. Dietary choline decreased lipid content in whole body and tissues. Highest TAG and cholesterol concentrations in serum and liver were recorded in fish fed the HFD. Similarly, compared with fish fed the HFD, dietary choline reduced vacuolar fat drops and ameliorated HFD-induced pathological changes in liver. Expression of genes of lipolysis pathways were up-regulated, and genes of lipogenesis down-regulated, by dietary choline compared with fish fed the HFD. Expression of *nfκb* and pro-inflammatory cytokines in liver and intestine was suppressed by choline supplementation, whereas expression of anti-inflammatory cytokines was promoted in fish fed choline-supplemented diets. In fish that received lipopolysaccharide to stimulate inflammatory responses, the expression of *nfκb* and pro-inflammatory cytokines in liver, intestine and kidney were all down-regulated by dietary choline compared with the HFD. Overall, the present study indicated that dietary choline had a lipid-lowering effect, which could protect the liver by regulating intrahepatic lipid metabolism, reducing lipid droplet accumulation and suppressing NFκB activation, consequently attenuating HFD-induced inflammation in *A. schlegelii*.

Lipids are important energy-dense macronutrients that are efficiently utilised by most fish species and, consequently, there is a tendency to use high-fat diets (HFD) in intensive aquaculture due to the protein-sparing and growth-promoting effects of dietary lipid^(1,2)^. However, HFD commonly cause excess fat accumulation in liver or visceral fat tissue in farmed fish, leading to liver injury and lipid metabolism disorders^(3–8)^. Similar results have been reported in mammals, indicating that excess dietary fat can cause injury in the liver and various other tissues and organs^(9–13)^. HFD-induced obesity in rodents has shown conclusively that hepatic steatosis is associated with a state of chronic hepatic inflammation^(14)^. Recently, studies demonstrated that HFD could impair lipid homoeostasis and induce inflammatory responses in several marine fish species, including black seabream (*Acanthopagrus schlegelii*), blunt snout bream (*Megalobrama amblycephala*) and large yellow croaker (*Larimichthys crocea*)^(4,7,9,15)^. It is generally accepted that excess fat deposition is associated with altered tissue lipid metabolism, including lipogenesis and lipolysis. Previous studies demonstrated that fat deposition could be decreased by down-regulating the expression of lipogenesis pathway genes such as sterol regulatory element-binding protein-1(*srebp-1*), acetyl-CoA carboxylase α (*accα*) and fatty acid synthase (*fas*), and/or up-regulation of lipolysis pathway genes such as PPARα (*pparα*), hormone-sensitive lipase (*hsl*) and carnitine palmitoyltransferase 1A (*cpt1a*)^(16–19)^. In addition, adiponectin, a hormone involved in the regulation of glucose metabolism and fatty acid breakdown in mammals, could lower intracellular lipid content^(20)^. Therefore, dietary supplements that can regulate lipid metabolism or adiponectin and, consequently, reduce excess lipid deposition, alleviate hepatic steatosis and attenuate inflammation response would be highly beneficial.

Choline has been shown to be an essential vitamin for fish, playing a vital role in maintaining cell structure and lipid transport in and out of the cells^(21,22)^. It is well known that choline is a key component of both phosphatidylcholine and acetylcholine, a neurotransmitter^(23)^. Recent studies reported that dietary choline supplementation affected hepatic transport and lipid deposition in various fish species, which suggested that dietary choline could reduce hepatic lipid content and influence expression of lipid metabolism genes^(23–26)^. Moreover, previous studies demonstrated that dietary choline could modulate immune responses by reducing expression of pro-inflammatory biomarkers such as TNF-α (*tnfα*), IL-1β (*il-1β*) and NFκB (*nfκb*), and up-regulating mRNA expression of anti-inflammatory cytokine IL-10 (*il-10*) and transforming growth factor β-1 (*tgfβ-1*) in vertebrates including fish^(27–29)^. However, there are few studies investigating the possible mechanisms whereby dietary choline affects lipid metabolism and subsequently reduces inflammatory response.

Lipopolysaccharide (LPS) is a structural component of the outer membrane of Gram-negative bacteria and one of the most effective stimulators of the immune system, and has been widely used as an experimental model for bacterial infection in animals^(4,28,30–33)^. Furthermore, the LPS inflammation model has been commonly used to evaluate acute-phase responses and the release of pro-inflammatory cytokines through the activation of *nfκb*^(4,34)^. NFκB is a transcription factor belonging to the ‘Rel’ family that represents a crucial intracellular signal transduction system involved in several inflammatory responses, through interaction with the inhibitory κB (IκB) proteins^(35)^. Activation of NFκB promotes the expression of inflammatory molecules, such as IL-6, IL-8 and TNFα^(36)^. Hence, NFκB is a key nuclear transcription factor tightly linked to the inflammatory response.

Black seabream (*A. schlegelii*) is a very popular and commercially important marine fish species cultured in China, Japan, Korea and other countries in Southeast Asia, and has been regarded as an excellent aquaculture species for intensive culture since it exhibits rapid growth, high disease resistance, and can tolerate a wide range of environmental conditions^(7)^. Besides, black seabream is a validated experimental model for HFD-induced inflammation as confirmed previously^(7)^. The production of high-quality fish for human consumption requires healthy fish and, therefore, improving fish health is a priority in aquaculture. In the present study, we aimed to investigate how HFD can affect lipid metabolism and cause inflammation by exploring the impacts of dietary choline, supplemented to HFD, as a mechanism to attenuate HFD-induced inflammatory responses.

## Materials and methods

### Ethics statement

Animal experimentation within the present study was conducted in accordance with the Animal Research Institute Committee guidelines of Ningbo University, China and approved by the Committee of the Animal Research Institute, Ningbo University, China.

### Experimental design and diet preparation

Five isonitrogenous (about 42 % crude protein) experimental diets with two levels of lipid (about 11 % and about 17 % crude lipid) were formulated with the diets containing the higher lipid level supplemented with graded levels of choline (Sinopharm Chemical Reagent Co., Ltd). The diets were termed: control, low-fat diet; HFD, high-fat diet; HFD + C1, HFD plus choline (3 g/kg dry diet); HFD + C2, HFD plus choline (6 g/kg dry diet); HFD + C3, HFD plus choline (12 g/kg dry diet) (Table 1). Fishmeal, soyabean protein concentrate, soyabean meal and wheat flour were used as protein sources, with fish oil, palmitic acid and soyabean lecithin used as the main lipid sources. All ingredients were purchased from Ningbo Tech-Bank Feed Co. Ltd. The experimental diets were produced according to the method described in detail previously^(7)^. Briefly, the ground ingredients were mixed in a Hobart type mixer and cold-extruded pellets produced (F-26; Machine Factory of South China University of Technology) with pellet strands cut into uniform sizes (2 and 4 mm diameter pellets) (G-250; Machine Factory of South China University of Technology). Pellets were steamed for 30 min at 90°C, and then air-dried to approximately 10 % moisture, sealed in vacuum-packed bags and stored at −20°C until used in the feeding trial.

**Table 1.** Formulation and composition of the experimental diets (% DM)

	Diets				
	Control	HFD	HFD + C1	HFD + C2	HFD + C3
Ingredients					
Fish meal	26·000	26·000	26·000	26·000	26·000
Soyabean protein concentrate	10·000	10·000	10·000	10·000	10·000
Soyabean meal	20·000	20·000	20·000	20·000	20·000
Wheat flour	24·300	24·300	24·300	24·300	24·300
Fish oil	8·000	8·000	8·000	8·000	8·000
Palmitic acid	0·000	6·000	6·000	6·000	6·000
Soyabean lecithin	1·000	1·000	1·000	1·000	1·000
Vitamin premix*	0·500	0·500	0·500	0·500	0·500
Mineral premix*	2·000	2·000	2·000	2·000	2·000
Choline chloride	0·000	0·000	0·300	0·600	1·200
Ca(H_2_PO_4_)_2_	1·000	1·000	1·000	1·000	1·000
Cellulose	7·200	1·200	0·900	0·600	0·000
Proximate composition (%)					
DM	90·04	89·43	89·78	89·72	89·59
Crude protein	40·48	40·79	41·49	41·68	41·92
Crude lipid	10·82	16·75	16·42	16·37	16·39
Ash	9·30	9·45	9·44	9·41	9·40

HFD, high-fat diet; HFD + C1, HFD + choline (3 g/kg); HFD + C2, HFD + choline (6 g/kg); HFD + C3, HFD + choline (12 g/kg).

* The vitamin premix and mineral mixture were purchased from Ningbo Tech-Bank Feed Co. Ltd.

### Feeding trial and experimental conditions

Juvenile black seabream (initial weight 8·16 ± 0·01 g) were obtained from a local commercial hatchery at Xiangshan Bay, Ningbo, China. Prior to the experiment, the black seabream juveniles were acclimatised for 2 weeks and fed with a commercial diet (45 % dietary protein, 12 % crude lipid; Ningbo Tech-Bank Corp.). A completely randomised trial design was implemented. Briefly, a total of 450 black seabream juveniles were randomly allocated to fifteen floating net cages (1·5 m × 1·5 m × 2·0 m) corresponding to triplicate cages of the five dietary treatments. Fish were hand-fed to apparent satiation twice daily at 07.00 and 17.00 hours over 8 weeks. During the experimental period, physico-chemical conditions including temperature (26·6–30·7°C), salinity (25·53–27·86 ‰), dissolved O_2_ (4·7–6·8 mg/l) and pH (8·0–8·1 mg/l) were monitored daily (YSI Proplus; YSI).

### Sample collection

At the end of the feeding trial, fish were sampled 24 h after the last feed, with all fish (other than six fish used for the LPS challenge test) anaesthetised with tricaine methane sulfonate (MS-222). All fish in each cage were weighed and counted to determine weight gain, specific growth rate, feed efficiency and survival. Five fish from each cage (fifteen per treatment) were pooled (*n* 3) and used for proximate composition of the whole body. Liver samples were collected and pooled from a further three fish per cage (*n* 3) and stored at −80°C prior to analysis of adiponectin, TAG and cholesterol content. Liver and intestine samples were also rapidly collected from five fish in each cage and stored at −80°C prior to analysis of gene expression (pools of five fish per cage, *n* 3). The liver was collected from one fish per cage into 4 % paraformaldehyde for histological analysis. Blood samples were taken from the caudal vein of eight fish per cage using 1·5 ml syringes.

### Proximate composition analysis

Crude protein, crude lipid, moisture and ash contents of diets as well as the lipid content of whole fish, muscle and liver were determined according to the methods of the Association of Official Analytical Chemists^(37)^. Briefly, crude protein content was determined via the Dumas combustion method with a protein analyser (FP-528; Leco). Moisture was determined by drying the samples to a constant weight at 105°C. Crude lipid contents were determined by Soxhlet extraction using diethyl ether (Soxtec System HT6; Tecator). Ash contents were determined using a muffle furnace at 550°C for 8 h.

### Assay of serum and hepatic biochemical indices

Blood was assayed within 24 h of collection after storage at 4°C, with serum collected by centrifugation at 956 ***g*** for 10 min at 4°C. Serum biochemical indices including total protein, albumin, TAG, cholesterol, glucose contents and the activities of alkaline phosphatase, aspartate aminotransferase (AST) and alanine aminotransferase (ALT) were measured by automatic biochemical analyser (Selectra Pro-M 13-7476) according to the manufacturer's instructions (Nanjing Jiancheng Bioengineering Institute).

Liver samples were homogenised in nine volumes (w/v) of ice-cold physiological saline (0·89 %; w/v), and then centrifuged as above. The contents of adiponectin (Shanghai Jiancheng Bioengineering Institute), TAG and cholesterol (Nanjing Qiaodu Biotechnology Co., Ltd) were assayed using commercial kits according to the manufacturer's instructions by Multiskan spectrum (Thermo).

### Histological analysis of liver

Fresh liver tissue was fixed with 4 % paraformaldehyde before paraffin sections were prepared (Servicebio). Briefly, after fixation for at least 24 h, tissue samples were trimmed appropriately in a fume hood before being dehydrated in ethanol with concentration increasing incrementally from 75 % to 100 %. Liver samples were then embedded in paraffin and sliced into sections of 4 µm using a microtome. They were stained with haematoxylin and eosin and images were acquired under a microscope (Nikon Eclipse CI).

### Total RNA extraction, reverse transcription and real-time PCR

Gene expression was determined by reverse-transcriptase quantitative PCR (qPCR) as follows. Total RNA was extracted from tissues (liver, intestine and kidney) of juvenile black seabream using Trizol reagent (Takara) according to the manufacturer's instructions. Quantity and quality of isolated RNA were determined spectrophotometrically (Nanodrop 2000; Thermo Fisher Scientific) and on a 1·2 % denaturing agarose gel, respectively. The cDNA was prepared from 1000 ng of DNAase-treated RNA and synthesised using a PrimeScript™ RT Reagent Kit with gDNA Eraser (Perfect Real Time; Takara). The housekeeping gene *β-actin* was used as the reference gene after confirming its stability across the experimental treatments. Specific primers for the candidate genes *nfκb*, *il-1β*, *tnfα*, *tgfβ-1*, *il-10*, *accα*, *fas*, *srebp-1*, *lpl*, *cpt1a*, *hsl* and *pparα* used for qPCR were designed by Primer Premier 5.0 (Table 2). Amplification was performed using a quantitative thermal cycler (Lightcycler 96; Roche). The qPCR assays were performed in a total volume of 20 µl, containing 1·0 µl of each primer, 10 µl of 2× conc. SYBR Green I Master (Roche), 2 µl of 1/5 diluted cDNA and 6 µl diethyl pyrocarbonate (DEPC)-water. The thermal-cycling conditions used for qPCR were as follows: 95°C for 2 min, followed by forty-five cycles of 95°C for 10 s, 58°C for 10 s and 72°C for 20 s. Standard curves were generated using six different dilutions (in triplicate) of the cDNA samples, and the amplification efficiency was analysed using the equation *E* = 10^(−1/slope)^−1^(40)^. The amplification efficiencies of all genes were approximately equal and ranged from 87 to 109 %. All gene expression data were presented relative to the expression of the control group (reference group). The expression levels of the target genes were calculated using the 2 method as described by Livak & Schmittgen^(41)^.

**Table 2.** Primers for real-time quantitative PCR for inflammation related genes and *β-actin* of black seabream (*Acanthopagrus schlegelii*)

Gene	Nucleotide sequence (5′–3′)	Size (bp)	GenBank reference or publication	Functions
*il-1β*	Forward: CATCTGGAGGCGGTGAA	231	JQ973887	Pro-inflammation cytokine
	Reverse: CGGTTTTGGTGGGAGGA			
*tnfα*	Forward: GTCCTGCTGTTTGCTTGG	154	AY335443	Pro-inflammation cytokine
	Reverse: AATGGATGGCTGCCTTGG			
*nf-κb*	Forward: AGCCCAAGGCACTCTAGACA	154	MK922543	Nuclear transcription factor
	Reverse: GTTCTGGGCAGCTGTAGAGG			
*tgfβ-1*	Forward: GGGTTTCCAACTTCGGC	209	Xue *et al*.^(39)^	Anti-inflammation cytokine
	Reverse: TTGTGTCCGTGGAGCGT			
*il-10*	Forward: TGTCAAACGGTTCCTTGCAG	172	MK922542	Anti-inflammation cytokine
	Reverse: GGCATCCTGGGCTTCTATCT			
*accα*	Forward: AGTAGCCTGATTCGTTGGT	154	KX066238	Lipogenesis pathway
	Reverse: AGTAGCCTGATTCGTTGGT			
*fas*	Forward: AAGAGCAGGGAGTGTTCGC	213	KX066240	Lipogenesis pathway
	Reverse: TGACGTGGTATTCAGCCGA			
*srebp*-*1*	Forward: TGGGGGTAGGAGTGAGTAG	247	KX066235	Lipogenesis pathway
	Reverse: GTGAAGGGTCAGTGTTGGA			
*cptla*	Forward: TGCTCCTACACACTATTCCCA	203	KX078572	Lipolysis pathway
	Reverse: CATCTGCTGCTCTATCTCCCG			
*hsl*	Forward: AGCAACTAAGCCCTCCCCATC	179	KX066236	Lipolysis pathway
	Reverse: TCTTCACCCAGTCCGACACAC			
*pparα*	Forward: ACGACGCTTTCCTCTTCCC	183	KX066234	Lipolysis pathway
	Reverse: GCCTCCCCCTGGTTTATTC			
*β-actin*	Forward: ACCCAGATCATGTTCGAGACC	212	Jiao *et al*.^(38)^	Housekeeping gene
	Reverse: ATGAGGTAGTCTGTGAGGTCG			

*tgfβ-1*, Transforming growth factor β-1; *accα*, acetyl-CoA carboxylase α; *fas*, fatty acid synthase; *srebp-1*, sterol regulatory element-binding protein-1; *cpt1a*, carnitine palmitoyltransferase 1a; *hsl*, hormone-sensitive lipase.

### Lipopolysaccharide injection and sampling

After the 8-week feeding trial, six fish in each cage were randomly collected for LPS challenge to intensify inflammatory responses. LPS (*Escherichia coli* 055:B5; Sigma-Aldrich) was dissolved in sterile PBS (pH = 7·4) to a final concentration of 0·5 mg/ml. Fish from the HFD, HFD + C1, HFD + C2 and HFD + C3 treatments were individually injected intraperitoneally with 0·2 ml LPS at a dose of 2·5 mg/kg body weight. As control, fish were injected individually with the same volume of sterile PBS. Liver, intestine and kidney were collected from all fish 24 h after injection (samples were pooled per cage; *n* 3 per dietary treatment), and snap-frozen in liquid N_2_ and stored at −80°C for later gene expression analysis of *nfκb*, *il-1β*, *tnfα*, *tgfβ-1* and *il-10*^(4,31,42,43)^.

### Statistical analysis

Results are presented as means with their standard errors (number of replicates as indicated). The relative gene expression results (qPCR analyses) were expressed as mean normalised ratios corresponding to the ratio between the copy number of the target gene and the copy number of the reference gene, *β-actin*. The homogeneity of variances (Levene's test) were checked prior to ANOVA followed by Tukey's honestly significant difference test at a significance level of *P* ≤ 0·05 (IBM SPSS Statistics 20).

## Results

### Growth performance, feed utilisation, survival and lipid content

**Fig. 1.**
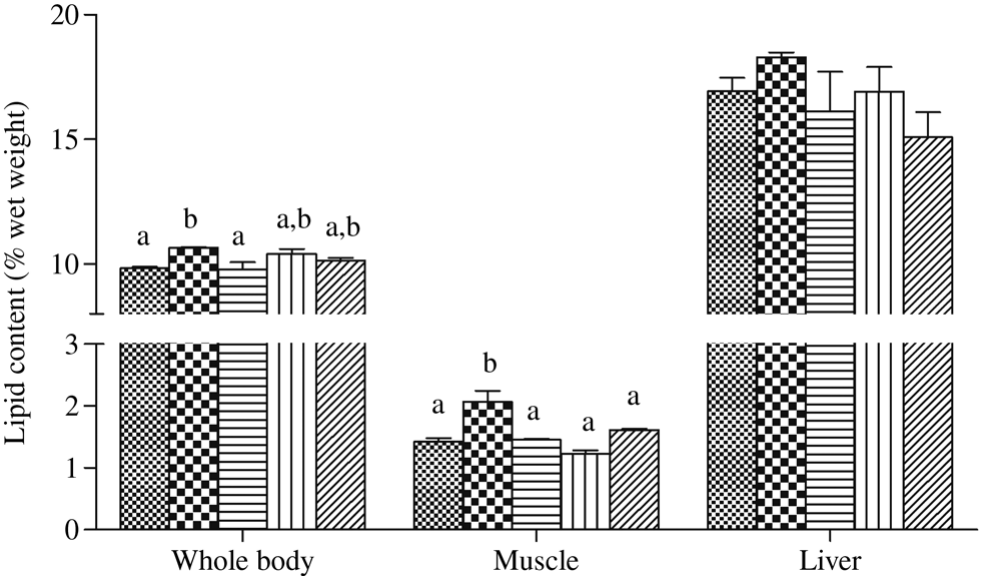
Whole body, muscle and liver lipid content of the juvenile black seabream (*Acanthopagrus schlegelii*) (% wet weight) fed the experimental diets (▒, control; 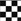, high-fat diet (HFD); ≡, HFD + choline (3 g/kg); ‖‖, HFD + choline (6 g/kg); ///, HFD + choline (12 g/kg)) for 8 weeks. Values are means (*n* 3), with their standard errors represented by vertical bars. ^a,b^Mean values with unlike letters within each tissue were significantly different (*P* < 0·05).

In the present study, no statistical differences were found in final body weight, weight gain, specific growth rate, feed efficiency or survival among the five groups (*P* > 0·05) (Table 3). However, fish fed with the HFD had significantly higher lipid content in whole body and muscle compared with the control group (*P* < 0·05). Furthermore, compared with the HFD treatment, the lipid content in muscle was significantly reduced by dietary choline supplementation (*P* < 0·05), and significantly lower lipid content in whole body was recorded in fish fed HFD + C1, but liver lipid content was not significantly affected (*P* > 0·05) (Fig. 1).

**Table 3.** Growth response, feed utilisation and survival of juvenile black seabream (*Acanthopagrus schlegelii*) fed the experimental diets for 8 weeks (Mean values with their standard errors; *n* 3)

	Diets										
	Control		HFD		HFD + C1		HFD + C2		HFD + C3		
Parameter	Mean	sem	Mean	sem	Mean	sem	Mean	sem	Mean	sem	ANOVA *P*
IBW (g)	8·14	0·01	8·16	0·01	8·16	0·01	8·16	0·01	8·16	0·01	0·933
FBW (g)	41·78	1·64	40·57	0·80	40·23	1·50	38·83	0·24	38·77	1·54	0·451
WG (%)*	412·91	19·50	397·39	9·22	393·25	17·69	376·14	2·61	375·34	18·37	0·406
SGR (%/d)†	2·92	0·07	2·86	0·03	2·85	0·07	2·79	0·01	2·78	0·07	0·410
FE (g/g)‡	0·60	0·03	0·57	0·01	0·56	0·03	0·54	0·00	0·54	0·03	0·330
Survival (%)§	95·56	1·11	98·89	1·11	96·67	1·92	95·56	2·94	95·56	2·22	0·716

HFD, high-fat diet; HFD + C1, HFD + choline (3 g/kg); HFD + C2, HFD + choline (6 g/kg); HFD + C3, HFD + choline (12 g/kg); IBW, initial body weight; FBW, final body weight; WG, weight gain; SGR, specific growth ratio; FE, feed efficiency.

* WG (%) = 100 × ((final body weight − initial body weight)/initial body weight).

† SGR (%/d) = 100 × ((Ln final body weight (g) − Ln initial body weight) (g)/d).

‡ FE = weight gain (g, wet weight)/feed consumed (g, dry weight).

§ Survival (%) = 100 × (final fish number/initial fish number).

### Serum and hepatic biochemical indices

**Fig. 2.**
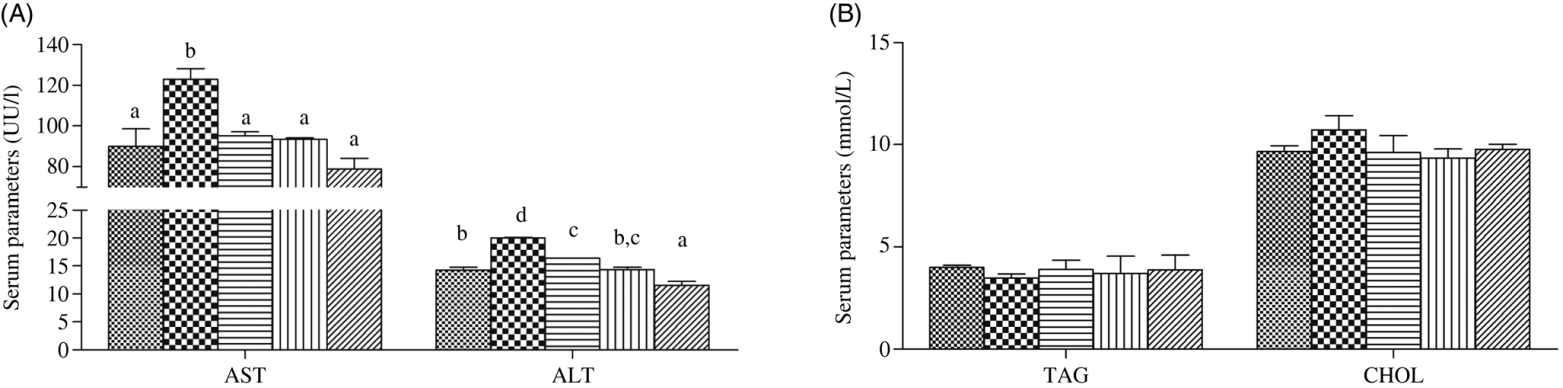
Serum parameters of juvenile black seabream (*Acanthopagrus schlegelii*) fed the experimental diets (▒, control; 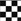, high-fat diet (HFD); ≡, HFD + choline (3 g/kg); ‖‖, HFD + choline (6 g/kg); ///, HFD + choline (12 g/kg)) for 8 weeks. Values are means (*n* 3), with their standard errors represented by vertical bars. ^a,b,c,d^Mean values with unlike letters within each serum parameter were significantly different (*P* < 0·05). AST, aspartate aminotransferase; ALT, alanine aminotransferase; CHOL, cholesterol.

**Fig. 3.**
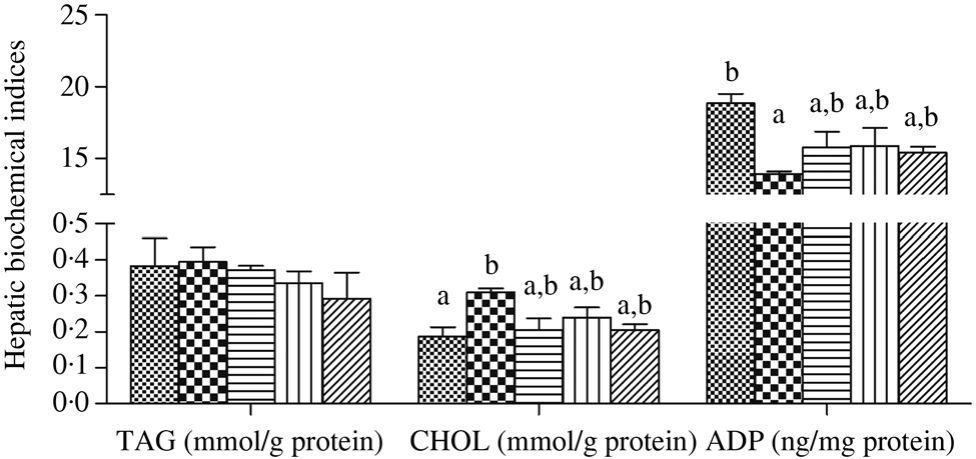
Hepatic biochemical indices of juvenile black seabream (*Acanthopagrus schlegelii*) fed the experimental diets (▒, control; 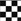, high-fat diet (HFD); ≡, HFD + choline (3 g/kg); ‖‖, HFD + choline (6 g/kg); ///, HFD + choline (12 g/kg)) for 8 weeks. Values are means (*n* 3), with their standard errors represented by vertical bars. ^a,b^Mean values with unlike letters within each hepatic index were significantly different (*P* < 0·05). CHOL, cholesterol; ADP, adiponectin.

In serum, the activities of AST and ALT were significantly higher in fish fed the HFD than in fish fed the other diets (*P* < 0·05), and dietary choline supplementation significantly decreased AST and ALT activities in fish fed the HFD (*P* < 0·05) (Fig. 2(A)). In contrast, no significant differences were found in serum TAG and cholesterol contents among all treatments (*P* > 0·05) (Fig. 2(B)). In liver, the cholesterol concentration was significantly higher in fish fed the HFD than in the control group, but was reduced in fish fed diets HFD + C1, HFD + C2 and HFD + C3 (*P* < 0·05). Contrasting results were found for hepatic adiponectin, with significantly lower concentration recorded in fish fed the HFD compared with the control group (*P* < 0·05), and there was a clear trend for dietary choline to increase adiponectin compared with the HFD group, although no statistical differences were found (*P* > 0·05) (Fig. 3).

### Hepatic histological analysis

**Fig. 4.**
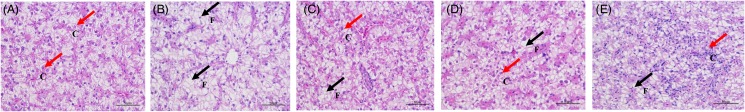
Paraffin section of liver in juvenile black seabream (*Acanthopagrus schlegelii*). The liver section was stained with haematoxylin and eosin to enhance the contrast (400×). (A) Paraffin section of liver in the control group; (B) paraffin section of liver in the high-fat diet (HFD) group; (C) paraffin section of liver in the HFD + choline (3 g/kg) group; (D) paraffin section of liver in the HFD + choline (6 g/kg) group; (E) paraffin section of liver in the HFD + choline (12 g/kg) group. C, cell nucleus; F, fat drop.

In fish fed the control diet, hepatocyte shape and structure were regular and normal, the nucleus with nucleolus was spherical, and basically in the middle of cells (Fig. 4(A)). In fish fed the HFD, hepatocyte nucleoli were vacuolar and had mostly disappeared, the nucleus and other organelles had lysed and liquefied to form large cysts, and cells contained many large vacuolar fat drops (Fig. 4(B)). In fish fed HFD + C1, HFD + C2 and HFD + C3, the shapes of some cells were regular and parts of the cell structure remained normal, some of the nuclei with nucleoli were spherical, and vacuolar fat drops fewer and smaller compared with the HFD group, suggesting that dietary choline prevented or reduced the cell damage caused by the HFD (Fig. 4(C)–(E)).

### Lipogenesis and lipolysis pathway key markers

**Fig. 5.**
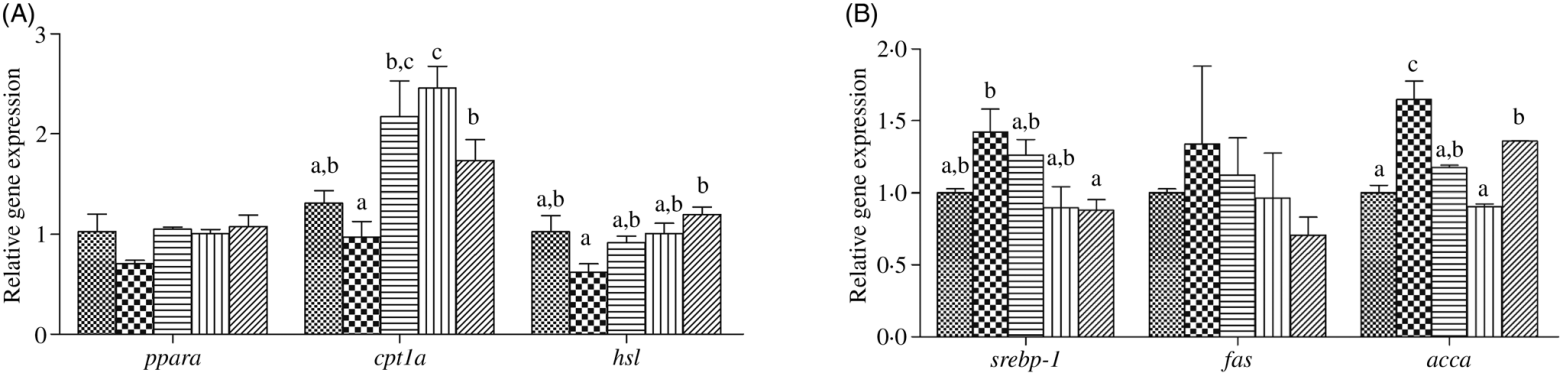
Lipid metabolism gene expression in liver of juvenile black seabream (*Acanthopagrus schlegelii*) fed the experimental diets (▒, control; 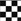, high-fat diet (HFD); ≡, HFD + choline (3 g/kg); ‖‖, HFD + choline (6 g/kg); ///, HFD + choline (12 g/kg)) for 8 weeks. The control was used as the reference group, and the mRNA expression levels of target genes were normalised relative to the expression of *β-actin*. Values are means (*n* 3), with standard errors represented by vertical bars. ^a,b,c^Mean values for each gene with unlike letters were significantly different (*P* < 0·05). *cpt1a*, Carnitine palmitoyltransferase 1a; *hsl*, hormone-sensitive lipase; *srebp-1*, sterol regulatory element-binding protein-1; *fas*, fatty acid synthase; *accα*, acetyl-CoA carboxylase α.

The hepatic expression of *cpt1a* was significantly up-regulated in fish fed the diets supplemented with choline supplementation compared with fish fed the HFD (*P* < 0·05). Similarly, *hsl* expression was significantly up-regulated in fish fed HFD + C3 compared with the HFD group (Fig. 5(A)). On the contrary, the hepatic expression levels of *srebp-1* and *accα* were significantly lower in fish fed the choline-supplemented diets compared with fish fed the HFD (*P* < 0·05) (Fig. 5(B)).

### Inflammatory markers after 8-week feeding trial

**Fig. 6.**
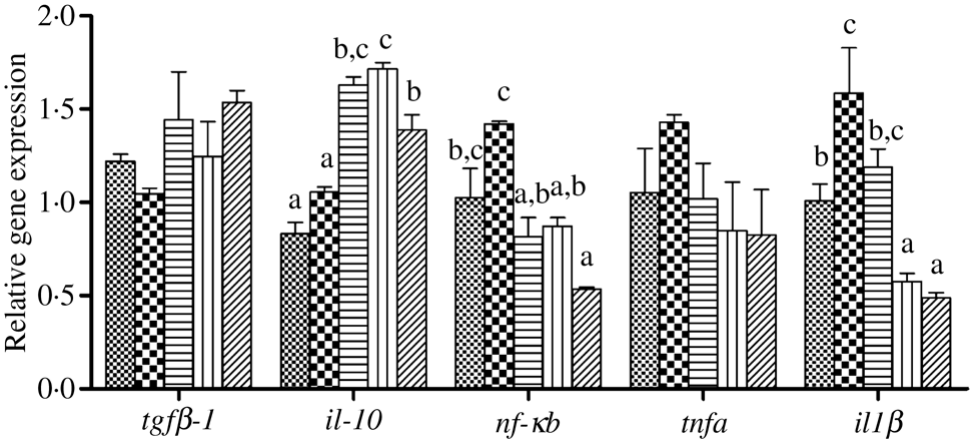
Inflammation gene expression in liver of juvenile black seabream (*Acanthopagrus schlegelii*) fed the experimental diets (▒, control; 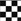, high-fat diet (HFD); ≡, HFD + choline (3 g/kg); ‖‖, HFD + choline (6 g/kg); ///, HFD + choline (12 g/kg)) for 8 weeks. The control was used as the reference group, and the mRNA expression levels of target genes were normalised relative to the expression of *β-actin*. Values are means (*n* 3), with standard errors represented by vertical bars. ^a,b,c^Mean values for each gene with unlike letters were significantly different (*P* < 0·05). *tgfβ-1*, Transforming growth factor β-1.

**Fig. 7.**
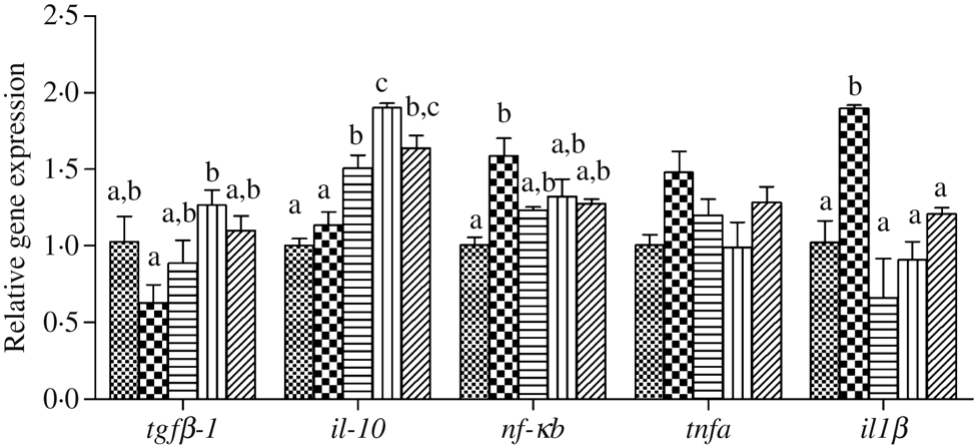
Inflammation gene expression in intestine of juvenile black seabream (*Acanthopagrus schlegelii*) fed the experimental diets (▒, control; 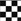, high-fat diet (HFD); ≡, HFD + choline (3 g/kg); ‖‖, HFD + choline (6 g/kg); ///, HFD + choline (12 g/kg)) for 8 weeks. The control was used as the reference group, and the mRNA expression levels of target genes were normalised relative to the expression of *β-actin*. Values are means (*n* 3), with standard errors represented by vertical bars. ^a,b,c^Mean values for each gene with unlike letters were significantly different (*P* < 0·05). *tgfβ-1*, Transforming growth factor β-1.

The expression levels of genes of the inflammatory response including nuclear transcription factor *nfκb*, pro-inflammatory cytokines *il-1β* and *tnfα* as well as anti-inflammatory cytokine *tgfβ-1* and *il-10* in liver and intestine are shown in Figs 6 and 7, respectively. In liver and intestine, the expression levels of *nfκb* and *il-1β* were significantly up-regulated in fish fed the HFD, and down-regulated in fish fed the diets supplemented with choline (*P* < 0·05). In contrast, no significant differences were found in *tnfα* mRNA expression (*P* > 0·05). The expression levels of *tgfβ-1* and *il-10* in liver and intestine were generally significantly up-regulated in fish fed the HFD with choline supplementation (*P* < 0·05), other than hepatic *tgfβ-1* mRNA expression, which was not statistically different (*P* > 0·05).

### Inflammatory markers after lipopolysaccharide injection

**Fig. 8.**
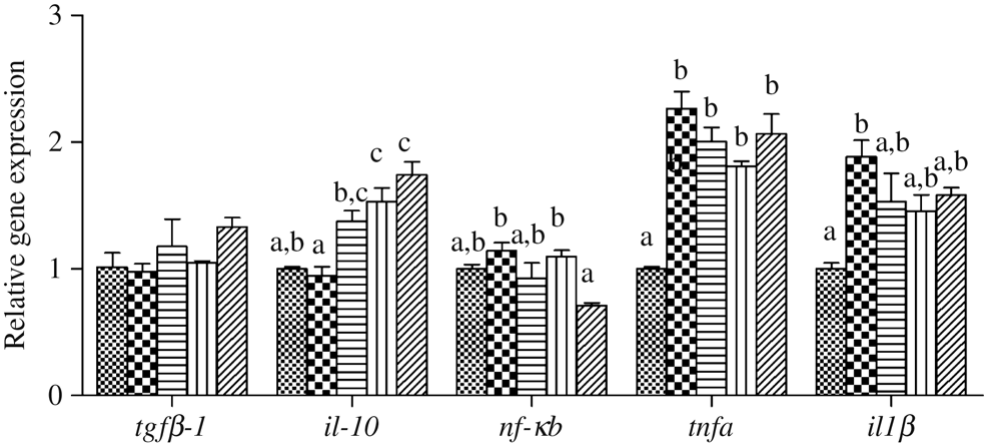
Effects of lipopolysaccharide injection for 24 h on inflammation gene expression in liver of juvenile black seabream (*Acanthopagrus schlegelii*) after feeding the 8-week experimental diets (▒, control; 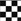, high-fat diet (HFD); ≡, HFD + choline (3 g/kg); ‖‖, HFD + choline (6 g/kg); ///, HFD + choline (12 g/kg)). The control was used as the reference group, and the mRNA expression levels of target genes were normalised relative to the expression of *β-actin*. Values are means (*n* 3), with standard errors represented by vertical bars. ^a,b,c^Mean values for each gene with unlike letters were significantly different (*P* < 0·05). *tgfβ-1*, Transforming growth factor β-1.

**Fig. 9.**
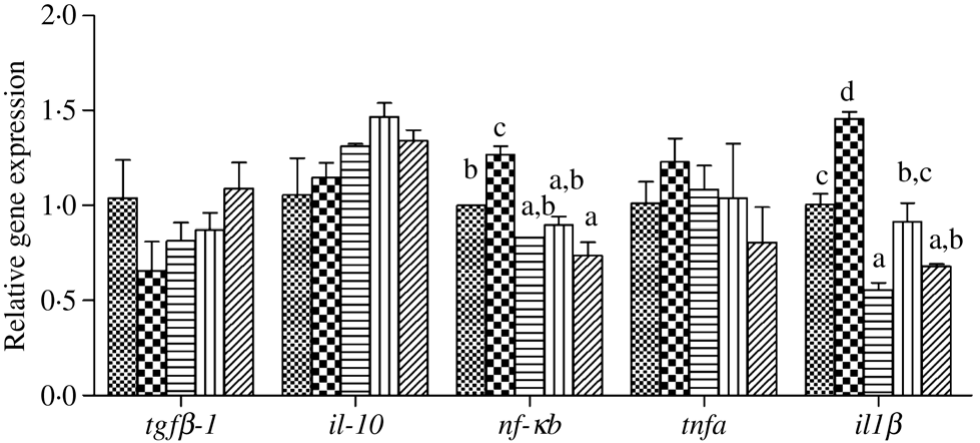
Effects of lipopolysaccharide injection for 24 h on inflammation gene expression in intestine of juvenile black seabream (*Acanthopagrus schlegelii*) after feeding the 8-week experimental diets (▒, control; 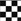, high-fat diet (HFD); ≡, HFD + choline (3 g/kg); ‖‖, HFD + choline (6 g/kg); ///, HFD + choline (12 g/kg)). The control was used as the reference group, and the mRNA expression levels of target genes were normalised relative to the expression of *β-actin*. Values are means (*n* 3), with standard errors represented by vertical bars. ^a,b,c,d^Mean values for each gene with unlike letters were significantly different (*P* < 0·05). *tgfβ-1*, Transforming growth factor β-1.

**Fig. 10.**
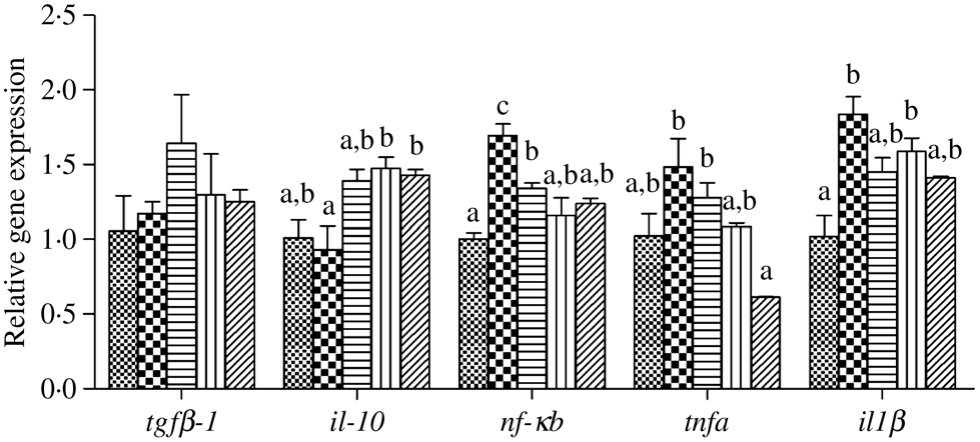
Effects of lipopolysaccharide injection for 24 h on inflammation gene expression in kidney of juvenile black seabream (*Acanthopagrus schlegelii*) after feeding the 8-week experimental diets (▒, control; 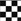, high-fat diet (HFD); ≡, HFD + choline (3 g/kg); ‖‖, HFD + choline (6 g/kg); ///, HFD + choline (12 g/kg)). The control was used as the reference group, and the mRNA expression levels of target genes were normalised relative to the expression of *β-actin*. Values are means (*n* 3), with standard errors represented by vertical bars. ^a,b,c^Mean values for each gene with unlike letters were significantly different (*P* < 0·05). *tgfβ-1*, Transforming growth factor β-1.

The expression levels of genes of the inflammatory response including nuclear transcription factor *nfκb*, pro-inflammatory cytokines *il-1β* and *tnfα* as well as anti-inflammatory cytokine *tgfβ-1* and *il-10* in liver, intestine and kidney of juvenile black seabream after LPS injection are presented in Figs 8–10, respectively. In all three tissues, the expression levels of *nfκb* were significantly higher in fish fed the HFD compared with fish fed the other diets, and down-regulated in fish fed the diets supplemented with choline (*P* < 0·05). Similarly, the pro-inflammatory cytokines *il-1β* and *tnfα* were decreased by dietary choline supplementation compared with fish fed the HFD (*P* < 0·05), although some differences were not statistically significant (*P* > 0·05). In contrast, expression levels of the anti-inflammatory cytokine *il-10* in liver and kidney were significantly lower in fish fed the HFD, and significantly up-regulated by choline supplementation (*P* < 0·05). However, although there was a trend of increasing expression of *il-10* in intestine in diets supplemented with choline, this was not significant (*P* > 0·05). Similar results were also recorded for *tgfβ-1* expression in liver, intestine and kidney, with expressions tending to increase in fish fed the choline-supplemented diets (HFD + C1, HFD + C2 and HFD + C3), but not statistically significant (*P* > 0·05).

## Discussion

Previous studies have demonstrated that choline is a vitamin for young vertebrates, it is the most abundant vitamin constituent in most fish feeds, and it can provide active methyl groups, which can participate in the anabolism of important physiological compounds such as methionine, phospholipids and carnitine^(22,24,25,28,29,44–46)^. Recent studies showed that adequate dietary supplementation of choline could improve growth performance and feed utilisation in various fish species^(24,25,45–47)^. In addition, weight gain increased significantly with increasing dietary choline supplementation when dietary lipid level was 11 %, and additional choline supplementation improved growth performance of *M. amblycephala* fed a HFD (15 % lipid)^(23,24)^. However, in the present study, graded levels of dietary choline (3, 6 or 12 g/kg) did not significantly affect the growth performance or feed utilisation of black seabream fed a HFD. The contrasting results could be explained by the higher dietary lipid level (16·5%) used in the present study. Additionally, it might be related to the duration of the trial, which was only 8 weeks in the present study. The impact of a HFD on growth was shown previously to be time-dependent in *M. amblycephala*^(4)^. Furthermore, few studies have been conducted on the effects of dietary choline in fish fed HFD. However, although growth performance and feed utilisation in black seabream were not affected by HFD supplemented with choline, the present study revealed that dietary choline had impacts on lipid deposition, lipid metabolism and inflammation response that gave insights to possible regulatory mechanisms.

AST and ALT are two important aminotransferases in fish that are often used as general indicators of vertebrate liver function^(23,48)^. General cellular damage occurring in hepatic steatosis and injury in mammals is usually monitored by analysing leakage of cellular enzymes like AST and ALT into the blood^(49,50)^, and similar mechanisms have also been confirmed in some fish species^(23,51,52)^. Significantly increased AST and ALT activities in serum were observed in fish fed diet HFD, consistent with a previous study in *M. amblycephala* fed HFD^(23)^. This suggested that there was a release of intracellular enzymes into the blood, indicating that possible damage to hepatocytes was induced by feeding HFD to black seabream. However, AST and ALT activities were reduced with increasing dietary choline in fish fed the HFD in the present study, demonstrating that dietary choline supplementation could mitigate the damage induced by a HFD in black seabream. Cholesterol and TAG levels in serum were unaffected by diet, whereas hepatic cholesterol and TAG concentrations were increased in fish fed the HFD, and decreased with choline supplementation, which was similar to results obtained in a previous study in Nile tilapia (*Oreochromis niloticus*)^(6)^. In addition, adiponectin plays a crucial role in hepatic lipid metabolism, with the beneficial effects of adiponectin in mammals being partially attributed to increased fatty acid oxidation in tissues such as liver and muscle^(53)^. In the present study, levels of adiponectin in liver were lowest in fish fed the HFD, and its level showed an increasing trend with dietary choline supplementation. This may indicate that choline supplementation to HFD treatments could reduce the risk of fat accumulation and hepatic steatosis in black seabream by reducing hepatic cholesterol and TAG, and restoring adiponectin concentration.

Choline has attracted attention as an important active substance in the body. Previous studies have confirmed that choline can act as an ‘anti-fatty liver' factor by preventing or reducing lipid deposition in the liver. Subsequently, numerous studies on choline have been carried out to further explore the mechanism of choline's anti-fatty liver effect, and provide insight into its important role in nutrient metabolism and regulation^(23–26,44,45)^. In the present study, the highest lipid contents in whole body and muscle were recorded in fish fed the HFD compared with fish fed the other diets, and muscle lipid contents were reduced in fish fed the HFD treatments supplemented with choline. Similar results have been reported previously in various fish species, confirming that dietary choline could reduce lipid content of fish^(23–26,45)^. The mechanism of dietary choline supplementation on lipid deposition caused by the HFD was further studied by investigating histopathological changes. The results indicated the damage that feeding a HFD could cause in the liver of black seabream, with the nucleus and other organelles lysed, forming large cysts, and the presence of many large vacuolar fat drops in hepatocytes, similar to results reported previously in mice^(54,55)^. The present study indicated that dietary choline could prevent this damage to the liver and/or promote almost complete recovery. These findings confirmed results from other fish species that demonstrated inverse correlations between dietary choline levels and hepatic lipid contents^(23,24,46)^.

To further explore the lipid-lowering mechanism of dietary choline, we herein analysed the relative expression of some hepatic genes involved in lipolysis (*pparα*, *cpt1a* and *hsl*) and lipogenesis (*srepb-1*, *fas*, and *accα*) pathways. It is believed that PPAR*α* can promote fatty acid β-oxidation, and modulate expression of genes encoding several mitochondrial fatty acid-catabolising enzymes^(56 )^, CPT1 is regarded as the main regulatory enzyme in fatty acid oxidation catalysing the conversion of cytosolic fatty acyl-CoA to fatty acyl-carnitine for entry into mitochondria^(57,58)^, and HSL is an important enzyme involved in lipolysis^(59)^. The present study indicated that, in fish fed HFD supplemented with choline, *cpt1a* and *hsl* expression levels were up-regulated compared with fish fed the HFD. This demonstrated that dietary choline could promote lipolysis and fatty acid β-oxidation by up-regulating key genes in these pathways. Moreover, FAS can catalyse *de novo* fatty acid synthesis^(60)^, SREBP-1 is a major regulator of fatty acid and lipid biosynthesis^(61)^, and ACCα is a cytosolic enzyme that controls the production of malonyl-CoA and thus plays an important role in the biosynthesis of long-chain fatty acids^(62–64)^. In the present study, the expression levels of *srebp-1* and *accα* showed a downward trend in the liver of fish fed the choline-supplemented diets. These results were consistent with previous studies in *A. schlegelii*, *Pseudosciaena crocea* and *O. niloticus*^(6,7,15)^. Hence, we conclude that dietary choline supplementation can reduce lipid deposition and alleviate hepatic steatosis through the regulation of lipid metabolism by up-regulating lipolysis and down-regulating lipogenesis pathway gene expression levels.

Previous studies revealed that HFD-induced lipid accumulation in the liver probably causes endoplasmic reticulum stress and accelerates the release of cytokines, thereby inducing inflammation^(54,65–67)^, which was confirmed in our previous study^(7)^. The nuclear transcription factor *nfκb* is a key upstream signalling molecule and when NF-κB is activated, it transfers into the nucleus and induces the expression of multiple inflammatory makers, including *tnf-α* and *il-1β*^(55,68)^. In the present study, the transcript expression levels of nuclear transcription factor *nfκb* was up-regulated in fish fed the HFD both in the liver and intestine compared with fish fed the control diet. Consequently, the pro-inflammatory cytokines *il-1β* and *tnfα* were also up-regulated in fish fed the HFD without choline supplementation, confirming previous reports in *M. amblycephala*^(23)^. The relative expression levels of *nfκb*, *il-1β* and *tnfα* were all decreased in fish fed the diets with choline supplementation. These results were generally consistent with recent studies, indicating that moderate levels of dietary choline could alleviate inflammation by modulating NF-κB signalling molecules^(26,28,29)^. Moreover, *tgfβ-1* and *il-10* are two common anti-inflammatory cytokines^(28)^ and they were up-regulated in the liver and intestine by choline supplementation in the present study. Similar results were obtained in other fish species^(28,29)^. Combined, these results indicated that dietary choline supplementation could reduce inflammatory responses. Hence, the present study demonstrated that fish fed the HFD caused lipid deposition, activated NFκB, and pro-inflammatory cytokines were released, thereby causing an inflammatory response. Dietary choline supplementation could attenuate inflammation by modulating NF-κB signalling molecules and increasing expression of anti-inflammation markers.

In order to further verify that dietary choline supplementation has the effect of relieving inflammation, the LPS challenge experiment was conducted to promote a strong inflammation response. In teleosts, it is well known that inflammatory challenges *in vivo* and *in vitro* are able to induce the expression of genes of various pro-inflammatory factors with rapid kinetics^(69)^. Recently, it reported that the LPS effect varied depending upon the cytokine, stimulating (*il-1β*), inhibiting (*tgfβ-1*) or ineffective (*tnf-α*)^(43)^. In the present study, the highest expression levels of *nfκb*, *il-1β* and *tnfα* in the liver, intestine and kidney were all recorded in fish fed the HFD 24 h after LPS injection, and all were lower in fish fed supplementary dietary choline. On the contrary, after LPS the lowest expression levels of *il-10* in the liver and kidney were found in fish fed the HFD, similar to results obtained in other fish species^(4,28,30–32)^. Likewise, *il-10* expression levels were up-regulated by dietary choline supplementation. However, no significant differences were found in *tgfβ-1* expression in any tissue, although there was an upward trend. Therefore, the LPS injection experiment generally confirmed that dietary choline supplementation had an effect of relieving inflammation by regulating inflammatory cytokines expression.

### Conclusion

**Fig. 11.**
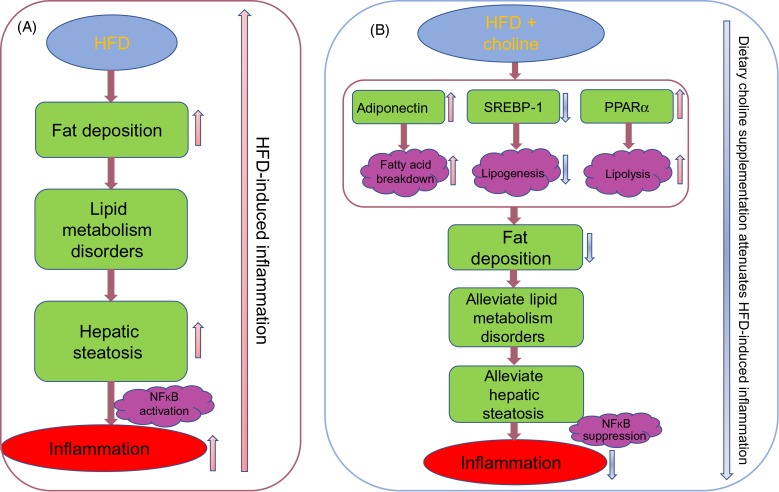
Mechanism of high-fat diet (HFD)-induced inflammation of black seabream (*Acanthopagrus schlegelii*) (A); mechanism of dietary choline supplementation attenuation of HFD-induced inflammation response in black seabream (B). Pink arrows represent increase/up-regulate, blue arrows present decrease/down-regulate. SREBP-1, sterol regulatory element-binding protein-1.

In conclusion, the present study provided further insight to the mechanism of the HFD-induced inflammatory response that results in lipid accumulation, hepatic steatosis and NFκB activation (Fig. 11(A)). Furthermore, the present study revealed that dietary choline supplementation attenuated the HFD-induced inflammatory response (Fig. 11(B)). Dietary choline supplementation could increase hepatic adiponectin content and expression of lipolysis pathway genes, and reduce expression of lipogenesis pathway genes, promoting a lipid-lowering effect, and restoring lipid metabolism balance, and reducing hepatic steatosis and, subsequently, attenuating inflammation by modulating NF-κB signalling molecules to suppress pro-inflammatory genes and increasing expression of anti-inflammatory genes.
